# Chromosome3D: reconstructing three-dimensional chromosomal structures from Hi-C interaction frequency data using distance geometry simulated annealing

**DOI:** 10.1186/s12864-016-3210-4

**Published:** 2016-11-07

**Authors:** Badri Adhikari, Tuan Trieu, Jianlin Cheng

**Affiliations:** Computer Science Department, University of Missouri, Columbia, MO 65211 USA

**Keywords:** Genome structure, Chromosome structure, Three-dimensional modelling, Distance geometry, Simulated annealing

## Abstract

**Background:**

Reconstructing three-dimensional structures of chromosomes is useful for visualizing their shapes in a cell and interpreting their function. In this work, we reconstruct chromosomal structures from Hi-C data by translating contact counts in Hi-C data into Euclidean distances between chromosomal regions and then satisfying these distances using a structure reconstruction method rigorously tested in the field of protein structure determination.

**Results:**

We first evaluate the robustness of the overall reconstruction algorithm on noisy simulated data at various levels of noise by comparing with some of the state-of-the-art reconstruction methods. Then, using simulated data, we validate that Spearman’s rank correlation coefficient between pairwise distances in the reconstructed chromosomal structures and the experimental chromosomal contact counts can be used to find optimum conversion rules for transforming interaction frequencies to wish distances. This strategy is then applied to real Hi-C data at chromosome level for optimal transformation of interaction frequencies to wish distances and for ranking and selecting structures. The chromosomal structures reconstructed from a real-world human Hi-C dataset by our method were validated by the known two-compartment feature of the human chromosome organization. We also show that our method is robust with respect to the change of the granularity of Hi-C data, and consistently produces similar structures at different chromosomal resolutions.

**Conclusion:**

Chromosome3D is a robust method of reconstructing chromosome three-dimensional models using distance restraints obtained from Hi-C interaction frequency data. It is available as a web application and as an open source tool at http://sysbio.rnet.missouri.edu/chromosome3d/.

**Electronic supplementary material:**

The online version of this article (doi:10.1186/s12864-016-3210-4) contains supplementary material, which is available to authorized users.

## Background

Three-dimensional (3D) chromosome structures provide insights into cellular processes such as the regulation of gene expression, DNA repair and replication and methylation. Traditionally, fluorescence in situ hybridization (FISH) is used to study the 3D organization of chromosomes and genomes [1–3]. However, due to the low throughput and low resolution of FISH data, it cannot be used to study the organization of chromosomes and genomes at a finer and larger scale. Recently, chromosome conformation capture techniques like Hi-C [4] and TCC [5] have emerged as powerful techniques to capture the proximity between chromosomal fragments and to study the 3D organization of chromosomes and genomes. The chromosomal contacts generated by Hi-C data can be used to infer 3D structures of chromosomes and genomes. A typical Hi-C experiment produces a matrix of interaction frequencies (IFs) between pairs of loci at a granularity defined in terms of resolution. An interaction frequency matrix is often termed as chromosomal contact matrix. The bigger the IF between two loci, the higher probability they are close in the 3D space.

Several methods have been developed to reconstruct 3D chromosome and genome structures from contact matrices [6–13]. Many of these are distance-based methods, which translate the IF values in a contact matrix into Euclidean distances, called wish-distances and then try to place the loci in three-dimensional space in order to satisfy these distances. The distance satisfaction problem is often formulated as a computational optimization problem. Since Hi-C contact matrices are obtained from a population of cells whose chromosomal structures may vary and the exact relationship between IFs and physical distances between loci is unknown, wish-distances often conflict with each other and cannot be satisfied accurately or completely. Moreover, because of conflicting distances, there is not always a unique solution to the optimization problem. As a result, some methods generate an ensemble of structures that conform with a contact matrix [5, 6, 8], while others produce one consensus structure for each matrix [7, 9–12].

Inspired by the computational techniques such as Crystallography & NMR System (CNS) suite [14–18] used for reconstructing protein structures from atom-atom distances measured by X-ray crystallography and nuclear magnetic resonance (NMR), in this work, we introduce a distance geometry simulated annealing (DGSA) based method to reconstruct three-dimensional chromosomal structures from the chromosomal wish distances derived from chromosomal interaction frequencies. In addition, we use Spearman’s rank correlation [11] to compare reconstructed structures with original input interaction frequencies in order to investigate which conversion rule parameters for converting contacts into distances yields better reconstructed models. We validated our method with simulated datasets of a yeast chromosome [7] and a regular helix structure [16], and further tested it on a real Hi-C dataset [17].

## Results and discussion

### Reconstruction using simulated datasets

**Fig. 1:**
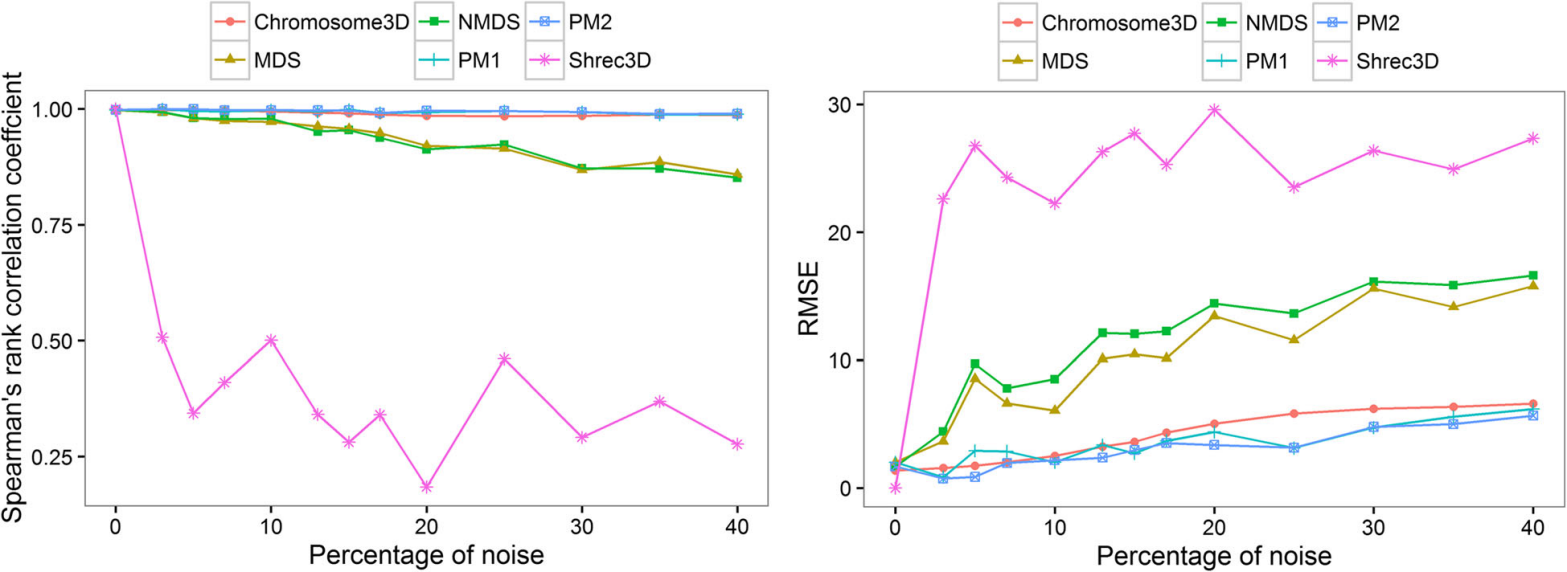
The accuracy of five methods on the simulated datasets. Correlation between true distances and reconstructed distances at different noise levels (*left*), and root mean square error (RMSE) between true distances and reconstructed distances at different noise levels (*right*)

In order to evaluate our method’s reconstruction on noisy datasets, we tested our method on the simulated datasets with noise, and compared its performance with five existing distance-based methods implemented in Pastis [9] and ShRec3D [12], including three classic multidimensional scaling methods (metric multidimensional scaling (MDS), non-metric multidimensional scaling (NMDS) and ShRec3D), two statistical methods using a Poisson distribution (PM1 and PM2). MDS directly infers the coordinates of points given their pairwise Euclidean distances. NMDS relies on the hypothesis that, if *IF*_*ij*_ > *IF*_*kl*_, then *d*_*ij*_ should be shorter than *d*_*kl*_, in order to derive a stress function to optimize. ShRec3D attempts to correct derived distances using shortest-path distance algorithm and then reconstructs 3D structures using classical MDS. PM1 and PM2 model IFs as Poisson random variables, and then try to maximize the likelihood of observing IFs. While PM1 needs a formula to convert the spatial distance to the Poisson intensity as prior knowledge, PM2 can automatically adjust the formula to infer structures that best explain the observed IFs. All five methods generate a consensus structure given a set of input data. We compare the robustness of methods to noise so that all methods were supplied with the true formula to convert IFs into wish distances. We reconstructed structures using all five methods and evaluated the reconstructed models by each of the five methods against the true structure using two measurements – (a) root mean square error (RMSE), and (b) Spearman’s rank correlation coefficient. For calculating RMSE, we simply compared the reconstructed models against the model of yeast chromosome 4. Similarly, Spearman’s rank correlation coefficient was computed between the distance matrix obtained from reconstructed three-dimensional structures and the distance matrix computed from the true structure. The comparison of all five methods, illustrated in Fig. 1, shows that our method performs similar to PM1 and PM2, which have higher accuracy than ShRec3D, MDS and NMDS. The reason for the bad performance of ShRec3D could be that its shortest-path algorithm failed to derive reasonable distances for reconstruction when short distances were affected by noise in our simulated dataset. Hence, in terms of accuracy of reconstruction, using simulated data we find that Chromosome3D is similar to other state-of-the-art methods like PM2 and ShRec3D. However, on real Hi-C data, we observed that PM2 failed to infer coordinates for all beads (several coordinates are ‘nan’ – see experimental data for detail) and ShRec3D failed to reconstruct structures for some chromosomes (all coordinates are ‘nan’ [see the “Availability of data and material” section]. Our method, on the other hand, always produces a reasonable structure with high reproducibility.

In addition, we also reconstructed the regular helix structure (having 100 points) used as benchmark dataset by Zou et al. [16] and compared our models with the models reconstructed by two other state-of-the-art methods, HSA [16] and ShRec3D [12]. Using Spearman’s rank correlation coefficient (SRCC) and Pearson’s correlation coefficient (PCC), we find that our method’s performance is similar to HSA and ShRec3D at 90, 70 and 25 % signal coverages [see Additional file 1]. Upon visualization using UCSF Chimera [18], we find that the reconstructed models appear similar for all three methods [see Additional file 2]. A high similarity between structures reconstructed by Chromosome3D and other methods validated our method’s reconstruction accuracy. However, in terms of speed, running reconstruction jobs for the regular helix structure of 100 points, we find that the state-of-the-art method, HSA, is around eight times slower than our method. For the speed test, we reconstructed models for the regular helix structure at 90, 70 and 25 % signal coverages using Chromosome3D and HSA. For each chromosome reconstruction, while Chromosome3D took on around 15 min, HSA took around 2 h (with all default parameters), on average. This slow speed of HSA method is probably because of its implementation and dependence on the R software platform and libraries. Because ShRec3D showed similar performance to our method and executed faster, we also compared our method with it on real Hi-C data (see sections below).

**Fig. 2:**
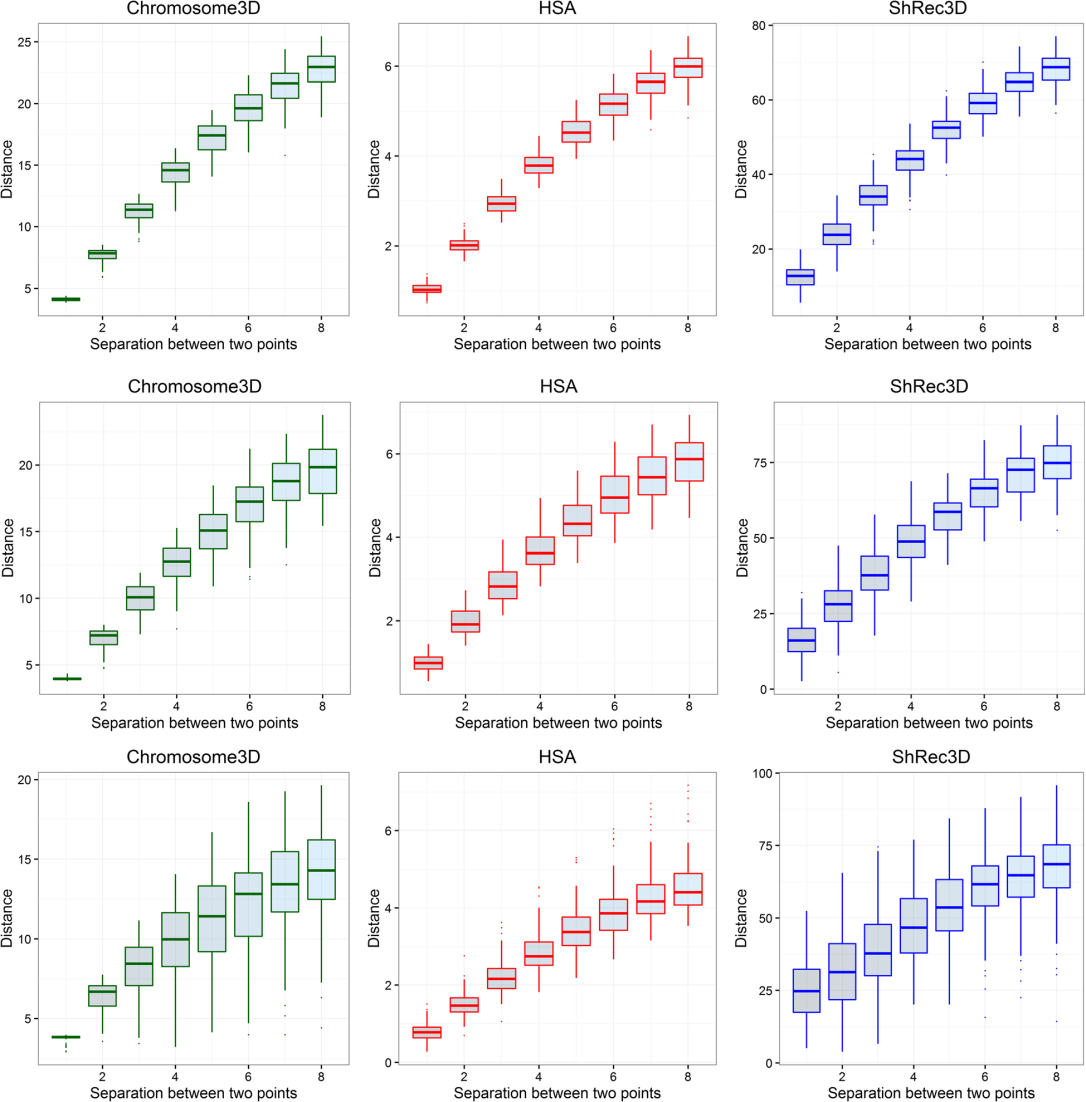
Distance distribution at various point separations. The distance distribution at sequence separations 1 through 8 for in models generated by Chromosome3D, HSA and ShRec3D for the regular helix structure at 90 % signal coverage (*top row*), 70 % signal coverage (*middle row*), and 25 % signal coverage (*last row*)

In addition to the assessing the reconstruction accuracy and speed, we also assessed our models to check if the models (in polymer representation) follow biophysical principles of distances. Specifically, we hypothesized that in the reconstructed models, all pairs of adjacent points should be at similar distance with low standard deviation and the distances and their deviations should increase as the separation between the points increases, at least for sequence separations that are a little higher than 1 like 2, 3, 4, etc. To test this, we observed the distribution of distances at each sequence separations from 1 to 8, and compared the distributions across our method, HSA and ShRec3D. Compared to HSA and ShRec3D, we find that our method has distributions more towards the ideal, i.e. adjacent points are always at a similar distance with almost no deviation and subsequent points have higher distances with gradually increasing deviations. In addition, we find that models generated by our method retain this distance distribution pattern irrespective of the noise in input interaction frequency. (See Fig. 2 for details).

### Reconstruction of chromosomal structures using real-world Hi-C data

**Fig. 3:**
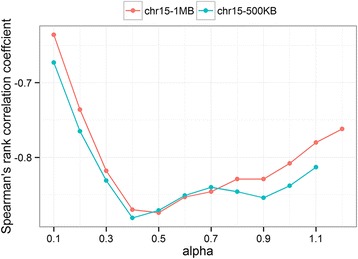
Alpha (α) in Eq. (1) versus SRCC values for chromosome 15 at 1 MB and 500 KB resolution. Best structures were obtained for alpha around 0.5


We applied Chromsome3D to reconstruct the structures for all 23 chromosomes of the human genome at resolutions 500 KB and 1 MB for a real-world Hi-C dataset of the cell line GM12878 [17] by converting IF matrices to wish distance matrices. This dataset had been normalized using a matrix balancing algorithm [17, 19]. Following the protocol to find the inverse relationship between IF matrix and wish-distances in Equation (1) discussed below, we find that the value of α around 0.5 yields the best SRCC values for all chromosomes at both resolutions (see Fig. 3), suggesting that an inverse square-root function produces models with best SRCC. Additional file 3 shows the visualizations of the structures of all 23 chromosomes at both resolutions and an additional movie file shows this in more detail [see Additional file 4].

Additional file 4: Video S1 Top structures for all 23 pairs of chromosomes (numbered sequentially) built using Chromosome3D and visualized using UCSF Chimera. For each chromosome two structures are shown side by side – a structure at 1 MB resolution on the left and a structure at 500 KB resolution on the right. (MP4 12371 kb)


Since the true structures of the chromosomes are unknown, we assessed the quality of reconstructed structures in three ways. First, we computed Spearman’s rank correlation coefficient between the distance matrix of reconstructed models and input IFs. Second, we compared the reconstructed structures at the two resolutions, 500 KB and 1 MB. Finally, we tested the two-compartment feature of chromosome discussed in [4]. Compared to the baseline extended structure, the substantial difference between Spearman’s rank correlation coefficients of the reconstructed models and those of extended models, at both resolutions (500 KB and 1 MB), suggests that the reconstructed models reliably satisfy the information captured by the IF data [see Additional file 5].

Furthermore, to make structures at 1 MB and 500 KB resolution directly comparable, we averaged every two adjacent points of structures at 500 KB resolution to obtain new structures with the same number of loci as the structures at 1 MB resolution. These new structures were then compared with the structures at 1 MB resolution, excluding Chromosomes 1, 2, 7, 9, 15, 18 whose number of loci at 500 KB resolution is not exactly the double number of loci at 1 MB resolution. We calculated the Spearman correlation and RMSEs between pairwise distances from the corresponding structures at 1 MB and 500 KB resolution (see Table 1). When calculating RMSEs, we rescaled structures to make the average pairwise distances of structures at two resolutions equal. This rescaling ensures that structures are in the same scale. We also computed RMSEs between 1 MB structures and the corresponding extended structures as references. The high values of Spearman’s rank correlation coefficients and low values of RMSEs of pairwise distances from the structures at the two resolutions demonstrate that the structures reconstructed by Chromsome3D at the two resolutions are similar.

**Table 1:** Correlation and RMSE between pairwise distances between some chromosomal structures at 1 M and 500 KB resolutions

Chromosome	Spearman’s rank correlation coefficient	RMSE
3	0.95	2.16 (8.92)
4	0.96	2.10 (5.25)
5	0.96	2.29 (6.50)
6	0.95	2.43 (9.05)
8	0.96	2.49 (6.60)
10	0.94	2.64 (8.14)
11	0.90	3.31 (10.16)
12	0.94	2.64 (8.22)
13	0.97	1.61 (6.81)
14	0.94	2.95 (8.30)
16	0.89	4.25 (10.80)
17	0.85	4.05 (9.85)
19	0.86	4.19 (13.36)
20	0.90	3.21 (9.31)
21	0.93	4.81 (5.93)
22	0.86	4.66 (7.58)
X	0.96	2.01 (6.08)

Numbers in parenthesis correspond to the RMSEs between 1 MB structures and the corresponding extended structures

**Fig. 4:**
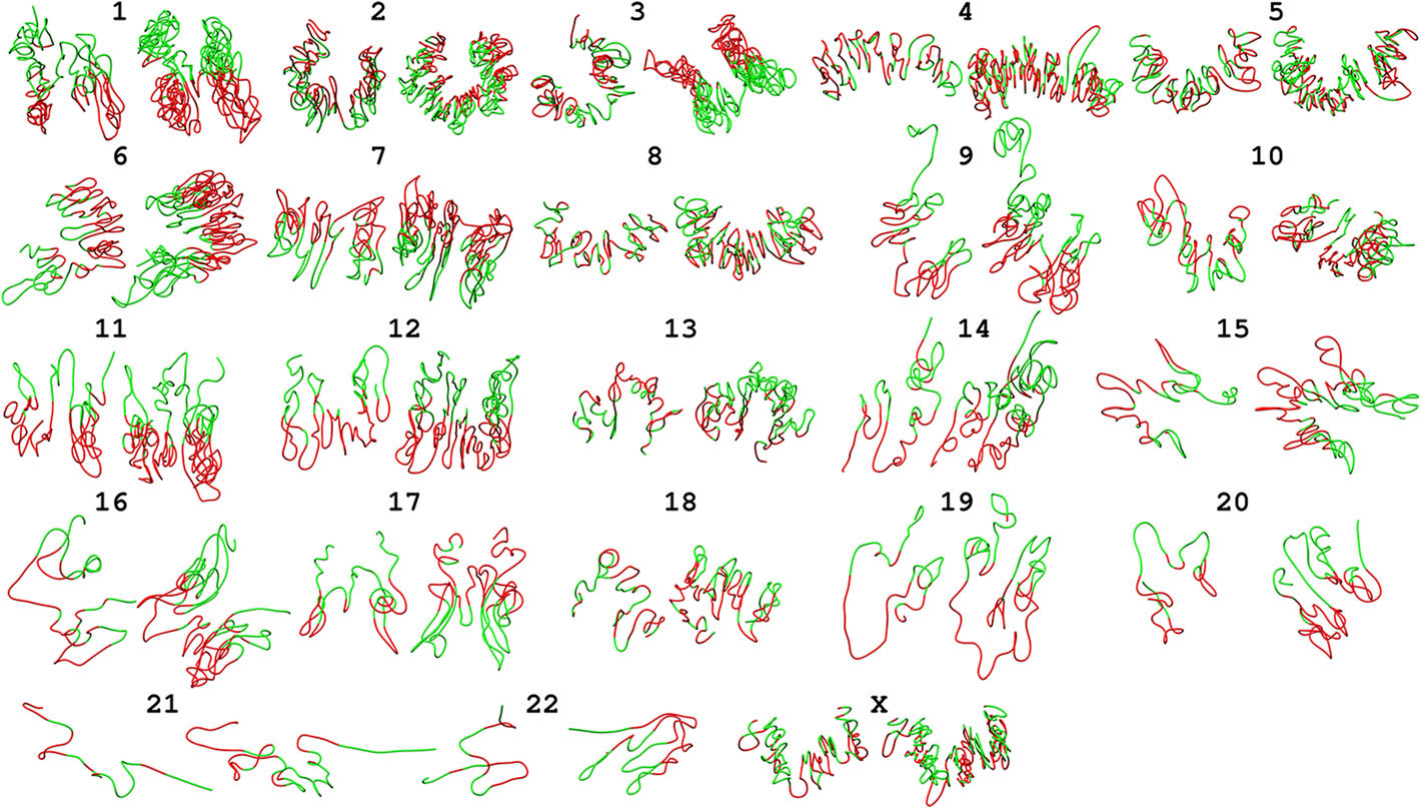
Two compartments of all 23 chromosomal structures at 1 MB resolution and 500 KB resolution. Compartments were obtained from the principal component analysis and colored in red and green, respectively


Our third assessment of the structures based on the two-compartment feature of chromosomes [4] is to check whether the chromosomes can be divided into two subspaces because regions in each subspaces preferentially interact with each other. We performed Principal Component Analysis (PCA) on the IF matrices to divide a chromosome into two regions – *euchromatin* and *heterochromatin* as in [4]. We visualized and colored regions of the two compartments with different colors to see if they are separable in the 3D structures as expected. The visualization in Fig. 4 and the additional movie file [see Additional file 6] shows that, except for the chromosome 21 and 22 at 1 MB resolution and for chromosome 22 in 500 KB resolution, the two compartments in chromosome structures are mostly separable, suggesting the partitioning of the two-chromatin partition feature of these chromosomes.

Additional file 6: Video S2 The two compartment features highlighted in top structures built using Chromosome3D for all 23 pairs of chromosomes (numbered sequentially) visualized using UCSF Chimera. For each chromosome two structures are shown side by side – a structure at 1 MB resolution on the left and a structure at 500 KB resolution on the right. (MP4 7997 kb)


### Comparison with other methods

**Fig. 5:**
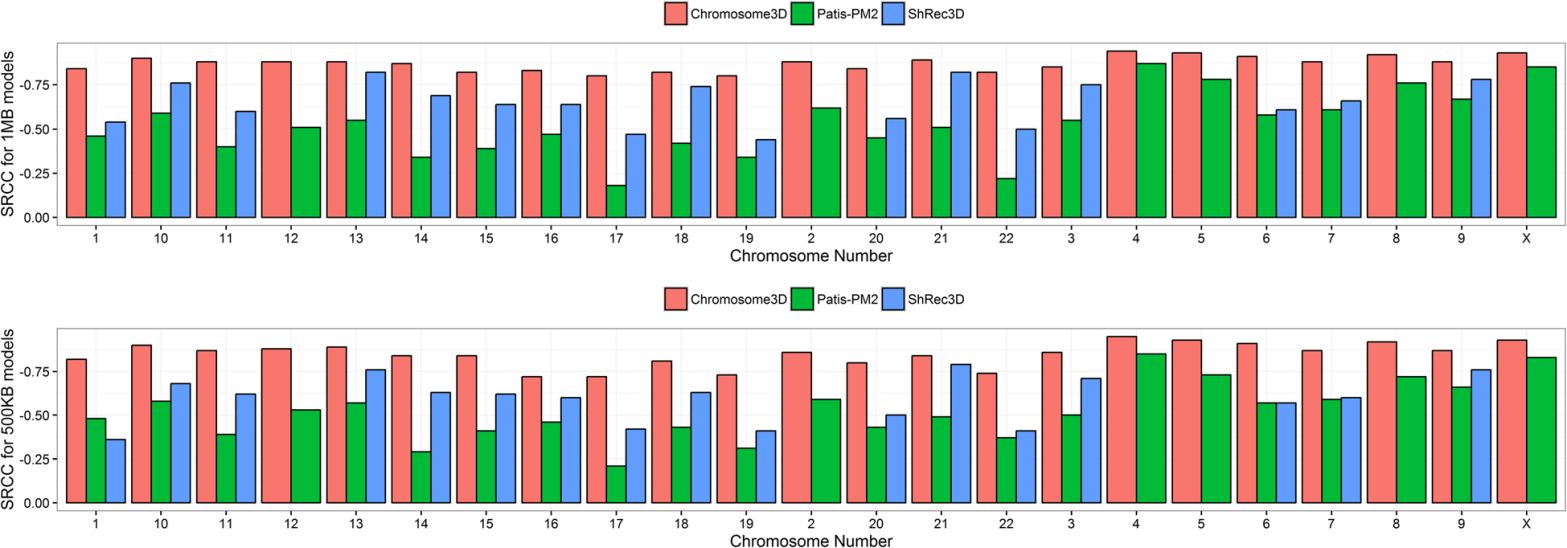
Comparison of the models generated by Chromosome3D, PM2 and ShRec3D for all 23 chromosomes on real Hi-C data at 1 MB (*top*) and 500 KB (*bottom*). Some plots have two bars (instead of three) because ShRec3D failed to generate models for those chromosomes

As a final part of our assessment, we reconstructed 3D chromosome models for the real Hi-C data at both resolutions (1 MB and 500 KB) using two existing state-of-the-art methods, PM2 (Pastis) [9] and ShRec3D [12], in order to compare with our method. We ignored the HSA [16] method because of its slow speed, considering the fact that our chromosome structures have up to 479 points. The average Spearman’s rank correlation coefficients between reconstructed models and input IF at 1 MB/500 KB reconstructed by Chromosome3D, PM2 and ShRec3D are −0.87/−0.85, −0.79/−0.78 and −0.65/−0.61 respectively. Comparison of the three methods is visualized in Fig. 5. Upon visualization, we find that the models generated by our method and PM2 are largely similar. For calculating SRCC values for the models generated by PM2 we ignored all the coordinates for which PM2 failed to infer coordinates (around 4 % of coordinates are ‘nan’ in the generated models). Besides having a poor reconstruction, ShRec3D, on the other hand, failed to generate models for some chromosomes. Finally, since the models generated by our method and PM2 visually looked similar (besides the SRCC evaluations), through visualization, we also compared the two-compartment features between the models. In general, we observed that both methods show similar regions as the compartments [see Additional file 7]. In conclusion, our method shows highly robust reconstructions comparable to the state-of-the-art methods with some advantages over existing methods. A limitation of our current implementation, however, is its inability to handle inputs having thousands of points. We plan to improve it in future by developing our own implementation of the DGSA optimization algorithm.

## Conclusions

The 3D conformation of a genome plays an important role in long-range gene regulation by bringing gene regulatory elements such as enhancers and transcription factor binding sites that are sequentially far away from a target gene closer to the gene spatially. The 3D genome conformation can also get the functionally related genes dispersed in different places in the genome sequence together to co-regulate. In order to facilitate applying 3D genome conformations to study this kind of long-range gene regulation, we introduced a distance geometry simulated annealing based method to reconstruct 3D chromosome structures from chromosomal contact data. The method was tested on simulated data and compared with existing methods and was shown to have fast reconstruction with accuracy at least as good as other stage-of-the-art methods. We also used the method to build chromosome structures of the cell line GM12878. These chromosome structures show the known two-compartment feature of the human chromosome and are stable to the change of resolution used to build structures. This result demonstrated that our method is useful for reconstructing chromosome structures to facilitate the study of the genome organization, the regulation of gene expression.

## Methods

**Fig. 6:**
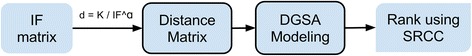
Reconstruction of chromosomal structures using distance geometry simulated annealing (DGSA)

We model chromosome structures as a string of beads in three-dimensional space where each bead represents a DNA fragment at a resolution (i.e. of a specific length). Our goal is then to place beads in the space such that: (a) the distances between beads inversely correlate with interaction frequencies between the beads, and (b) the distances between the beads optimally satisfy the wish distances. Our method, Chromosome3D, illustrated in Fig. 6, reconstructs three-dimensional structures from the interaction frequency (IF) matrix for each chromosome in three major steps. First we convert the input IF matrix to wish distance matrix using an inverse relationship function. In the past, the inverse relationship between IF data and physical distances had already been implemented as direct inverse relationship, 1/*IF* and as inverse cube-root relationship, 1/*IF* ^1/3^ [9]. Our experiments with many forms of available inverse functions showed that the precise relationship (for best reconstruction) depends upon the data at hand and precise conversion formula can be obtained through parameter tuning (discussed in detail in sections below). Second, for each input IF matrix, we build 20 structures using a Distance Geometry Simulated Annealing (DGSA) protocol. Finally, we compute Spearman’s rank correlation coefficients (SRCC) between the input interaction frequency matrix and the distance matrix of the reconstructed structures to rank the structures. Our experiments with computing SRCC showed that direct SRCC calculations are less accurate because of the presence of relatively large number of short-range values. Hence, for a reliable evaluation, we remove L/10 short-range values (L being the number of points to be modelled) for computing SRCC [see Additional file 8].

### Simulated dataset

In the absence of true chromosome structures for real-world Hi-C datasets, we tested our method with 13 artificial chromosomal contact datasets simulated from the theoretical 3D model of the yeast chromosome 4 [7] before applying it to the real-world Hi-C data. The chromosome is represented by 610 beads at 50 KB resolution. Interaction frequencies between beads were obtained using the formula $I{F}_{ij}=\frac{1}{d_{ij}}$, where *d*_*ij*_ is the Euclidean distance between beads *i* and *j* in the true model of the chromosome. IFs obtained by this formula were noise-free. The true value of *α* to convert IFs into wish distances (see Eq. 1) in this case is 1. And later, we tested our method to see if it could detect this value. For this testing, various levels of noise were introduced into the IF matrix [see Additional file 8 for details]. In addition to this realistic simulated data, for a more rigorous evaluation of Chromosome3D, we also reconstructed the regular helix structure introduced by Zou et al. [16] for benchmarking the HSA method, at various signal coverages and compared the reconstruction results with two other state-of-the-art methods.

### Conversion of interaction frequency to wish distance

In the absence of true chromosomal structures, a major challenge for any distance-based reconstruction method is to verify that the chosen conversion rule (to transform IF data to wish distance) is optimal. We hypothesize that appropriate conversion rule for a given dataset can be obtained by evaluating the reconstructed structures using SRCC. To test this, we reconstructed structures for the simulated data (structure of yeast chromosome 4), which is known to have a direct inverse relationship (with *α* set to 1), at various noise levels and find that the reconstructed structures having high SRCC values are those build using near direct inverse relationship.

**Fig. 7:**
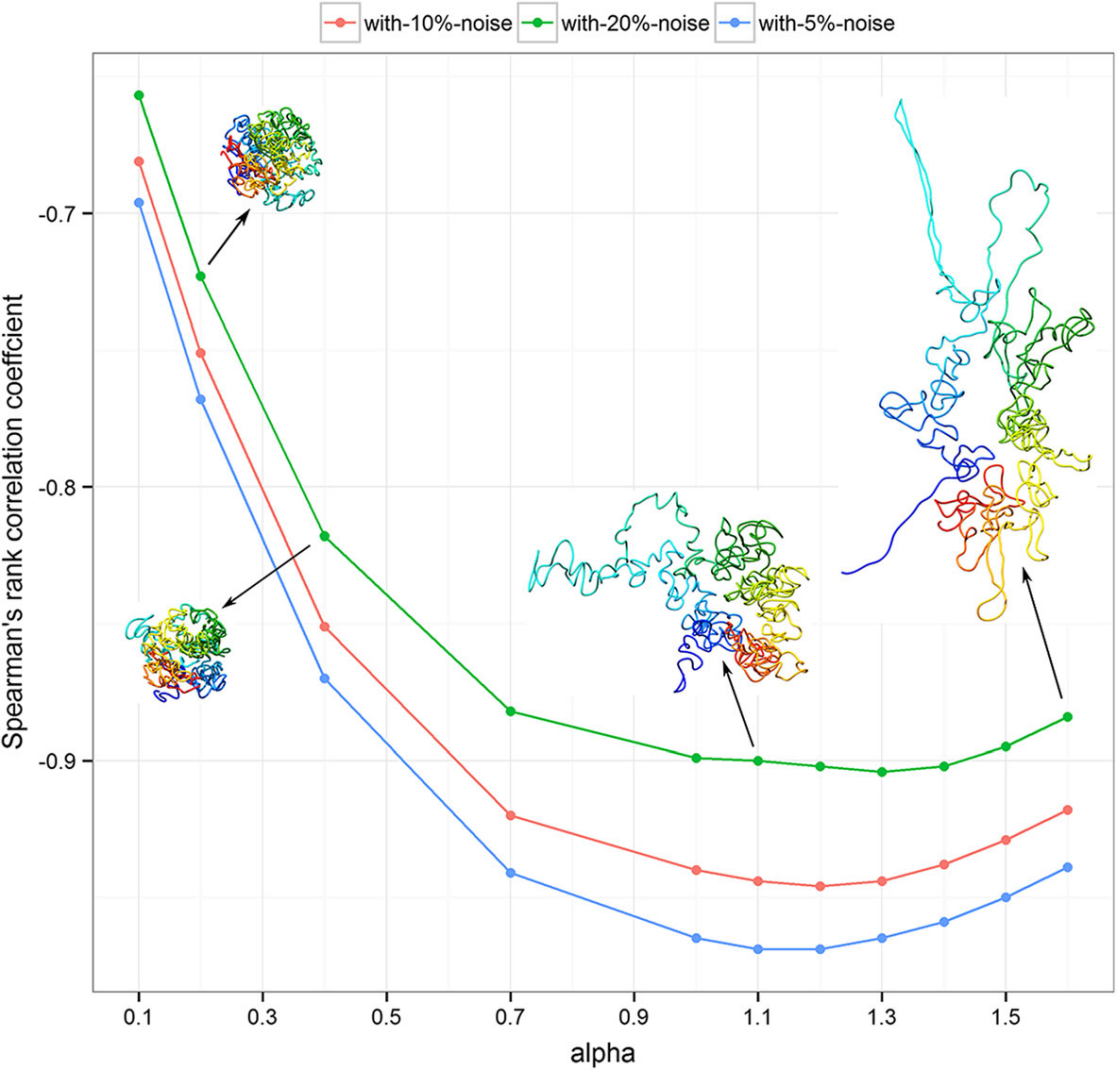
Quality of top structures at different values of *α* for simulated data with 5, 10 and 20 % noise. Spearman’s average rank correlation coefficient (against the input IF) is used to evaluate the quality of structures. The closer to − 1 the correlations are, the better structures. The structures are too compact for small α and too loose for large α

Specifically, each cell value *IF*_*ij*_ in the IF matrix is converted to distance using the following formula:

1 $$ {d}_{ij}=\frac{K}{{\left(I{F}_{ij}\right)}^{\alpha }/avg\left({\left(I{F}_{ij}\right)}^a\right)} $$

where *IF*_*ij*_ is the value in cell [i, j], K is the scaling constant, α is parameter to be tuned, and *avg*(*IF*_*ij*_) is the mean of all converted IF values. Each cell value is normalized by the mean, *avg*(*IF*_*ij*_), to make the conversion process invariant of the scale of input IF values. The scaling constant, K, controls the scale of the output structures, i.e., very small values result in structures with points very close to each other, and the structures, when visualized, look like a lump of points, and conversely, very large values overly relax the long-range distances, and the structures are stretched to look like extended structures. We find that the scaling constant K is dependent upon the implementation of reconstruction method, and for our DGSA implementation, values around 11 showed best reconstruction. Hence we used K equal to 11 for all of our experiments. The most important parameter, α, that controls the inverse relationship, needs to be tuned to maximize the SRCC of the reconstructed structures. Tuning *α* was also used in [11], where *α* was tuned to minimize the L1 error between predicted IFs and IFs from the input. However, in L1 error, outliers with large errors can dominate other terms and *α* can just be tuned to reduce the effect of these outliers. We found that correlation is a more robust measurement for the accuracy of predicted models. And it has been also used widely to assess the quality of predicted models [9, 12, 16, 20]. Using the simulated data with various levels of noise, where the actual relationship between the IF data and wish distances is a direct inverse, we reconstructed structures using various values of α and selected the top structures using Spearman’s rank correlation coefficients. The relationship between α and SRCC (see Fig. 7) shows that the best SRCC values are obtained for the conversions with α ~ 1.0 to 1.4, and this validates that relying on SRCC values to select conversion parameters is reasonably accurate.

### DGSA protocol for reconstructing structures

We use a customized version of the distance geometry simulated (DGSA) algorithm [21] originally proposed by Havel and Crippen [22] and implemented in the Crystallography & NMR System (CNS) suite for reconstruction. The wish-distances obtained from IF data are translated to protein carbon-alpha – carbon-alpha (Cα-Cα) distances in order to use them as restraints for the overall optimization process. We start the overall protocol with an extended structure of a chromosome where all chromosomal points (beads) are placed roughly in a line [see Additional file 8 for details]. Then, all Cα-Cα restraints are translated into upper and lower bounds on distances using set of inequalities that the distances have to satisfy. This gives two distance matrices: a matrix of lower bounds and, a matrix of upper bounds. To obtain a trial distance matrix, a distance matrix that gives rise to a single structure is generated by selecting a random distance that lies between the upper and lower restraints for each residue pair. By applying the law of cosines, the distance matrix is used to compute a metric matrix - a matrix of scalar products of position vectors of the atoms when the geometric centre is placed in the origin. The eigenvectors of the metric matrix gives the principal coordinates of the atoms. This starting structure is provided as input to molecular dynamics-based simulated annealing protocol to minimize the overall energy of the structure. Simulated annealing is carried out in two stages – 1000 steps of high temperature molecular dynamics annealing with starting temperature of 2000, followed by a slow-cool annealing. We defined the energy of a structure as the sum of all the physiochemical energies of a structure in conjunction with the restraint energy term, as defined below

2 $$ {E}_{total}={E}_{physio- chemical}+\left|{d}_{current- structure}-{d}_{wish}\right|{}^2 $$

where, *E*_*total*_ is the total energy of a structure, *E*_*physio* − *chemical*_ is the physiochemical energy of a structure that include terms like bond energies, dihedral angle energies and Van der Walls energies. The last term computes the squared error in the realization of our wish-distances. Final step of energy minimization is performed using 10 cycles of 15,000 steps of Limited-memory Broyden-Fletcher-Goldfarb-Shanno (LBFGS) minimization [23].

## Additional files

Additional file 1: Figure S1. Comparison of Chromosome3D with HSA and Shrec3D on the reconstruction of regular helical structure using Pearson’s correlation coefficient (PCC) and Spearman’s rank correlation coefficient (SRCC) at 25, 70 and 90 % signal coverages. PCC and SRCC are computed between the pairwise distance of the reconstructed models and the input interaction frequency matrix. (DOCX 118 kb)
Additional file 2: Figure S2. Reconstructed regular helical structure models reconstructed using Chromosome3D (top row), HSA (middle row) and Shrec3D (bottom row). The first column models (a) are the models reconstructed at 90 % signal coverage, second column models (b) at 70 %, and third column (c) at 25 % signal coverage. (DOCX 8090 kb)
Additional file 3: Figure S3. Top structures for all 23 pairs of chromosomes (numbered sequentially) visualized using UCSF Chimera. For each chromosome two structures are shown side by side – a structure at 1 MB resolution on the left and a structure at 500 KB resolution on the right. (DOCX 502 kb)
Additional file 5: Table S1. Spearman’s rank correlation coefficient between distances obtained from reconstructed structures and input IFs computed after filtering 0.1 L short-range values; the numbers in parenthesis are the SRCC values for extended models used for comparison. The last two columns report the execution time in hours and minutes. (DOCX 22 kb)
Additional file 7: Figure S4. The two compartment features highlighted in Chromosome 1 (left) and 2 (right) in the models reconstructed by Chromosome3D (top row) and PM2 (bottom row). (DOCX 761 kb)
Additional file 8: Supplementary sections on Methods. (DOCX 1850 kb)

## Abbreviations

3D: Three-dimensional
CNS: Crystallography & NMR System
DGSA: Distance geometry simulated annealing
FISH: Fluorescence in situ hybridization
IF: Interaction frequency
MDS: Metric multidimensional scaling
NMDS: Non-metric multidimensional scaling
NMR: Nuclear magnetic resonance
PCC: Pearson’s correlation coefficient
RMSE: Root mean square error
SRCC: Spearman’s rank correlation coefficient
