# Supplementary material for: Chromosome3D: reconstructing three-dimensional chromosomal structures from Hi-C interaction frequency data using distance geometry simulated annealing
Source: BMC Genomics. 2016 Nov 7;17:886. doi: 10.1186/s12864-016-3210-4 (PMC5100196; doi:10.1186/s12864-016-3210-4)
Supplement: Additional file 1: Figure S1. — Comparison of Chromosome3D with HSA and Shrec3D on the reconstruction of regular helical structure using Pearson’s correlation coefficient (PCC) and Spearman’s rank correlation coefficient (SRCC) at 25, 70 and 90 % signal coverages. PCC and SRCC are computed between the pairwise distance of the reconstructed models and the input interaction frequency matrix. (DOCX 118 kb) [file 12864_2016_3210_MOESM1_ESM.docx]

# **Chromosome3D: Reconstructing Three-Dimensional Chromosomal Structures from Hi-C Interaction Frequency Data using Distance Geometry Simulated Annealing**

### Badri Adhikari^§^**,** Tuan Trieu^§^**,** Jianlin Cheng*

Computer Science Department, University of Missouri, Columbia, Missouri, 65211, USA

*Corresponding author: [chengji@missouri.edu](mailto:chengji@missouri.edu)

^§^These authors contributed equally to this work


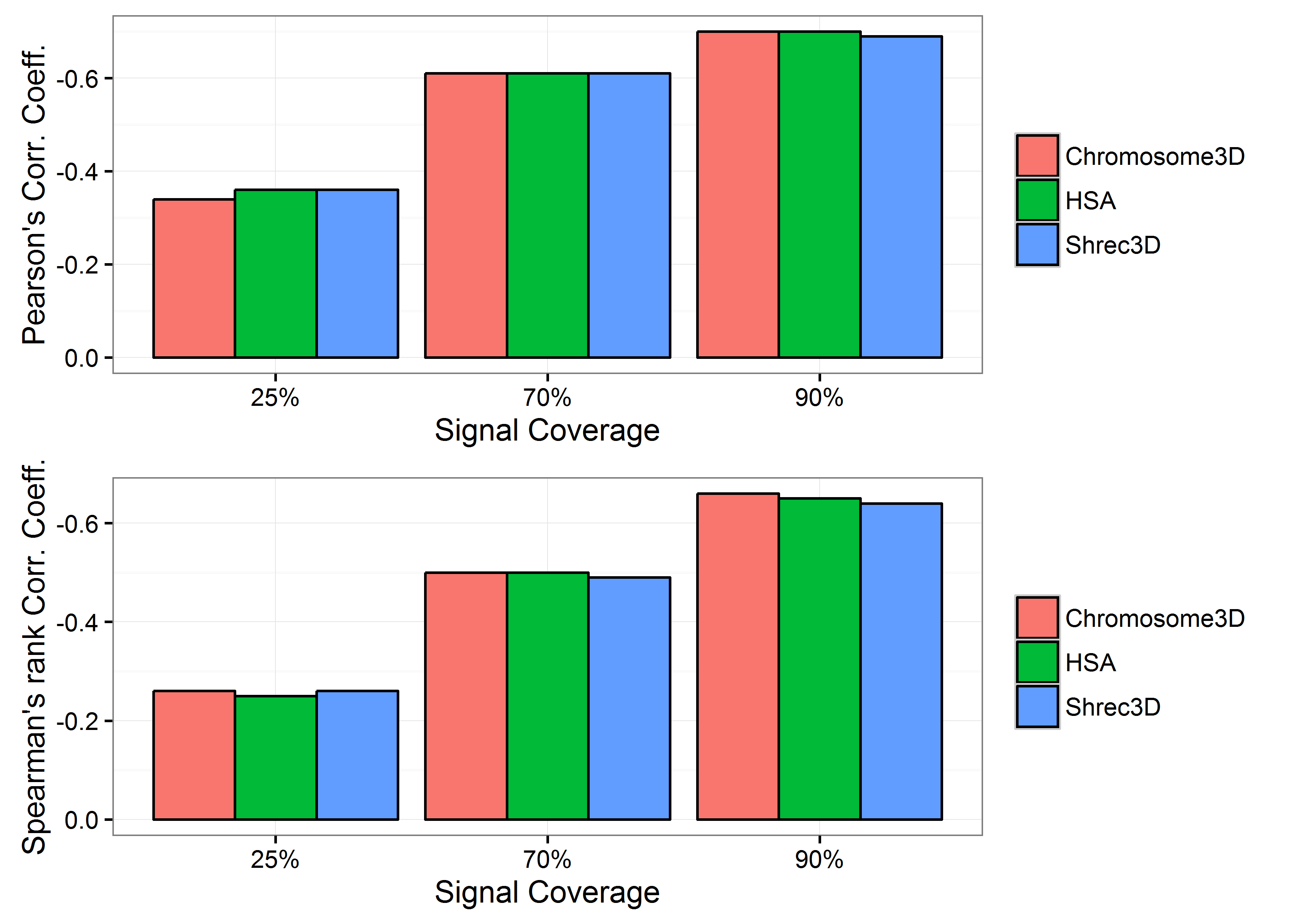


**Figure S1** Comparison of Chromosome3D with HSA and Shrec3D on the reconstruction of regular helical structure using Pearson’s correlation coefficient (PCC) and Spearman’s rank correlation coefficient (SRCC) at 25%, 70% and 90% signal coverages. PCC and SRCC are computed between the pairwise distance of the reconstructed models and the input interaction frequency matrix.
