# Supplementary material for: Chromosome3D: reconstructing three-dimensional chromosomal structures from Hi-C interaction frequency data using distance geometry simulated annealing
Source: BMC Genomics. 2016 Nov 7;17:886. doi: 10.1186/s12864-016-3210-4 (PMC5100196; doi:10.1186/s12864-016-3210-4)
Supplement: Additional file 2: Figure S2. — Reconstructed regular helical structure models reconstructed using Chromosome3D (top row), HSA (middle row) and Shrec3D (bottom row). The first column models (a) are the models reconstructed at 90 % signal coverage, second column models (b) at 70 %, and third column (c) at 25 % signal coverage. (DOCX 8090 kb) [file 12864_2016_3210_MOESM2_ESM.docx]

# **Chromosome3D: Reconstructing Three-Dimensional Chromosomal Structures from Hi-C Interaction Frequency Data using Distance Geometry Simulated Annealing**

### Badri Adhikari^§^**,** Tuan Trieu^§^**,** Jianlin Cheng*

Computer Science Department, University of Missouri, Columbia, Missouri, 65211, USA

*Corresponding author: [chengji@missouri.edu](mailto:chengji@missouri.edu)

^§^These authors contributed equally to this work


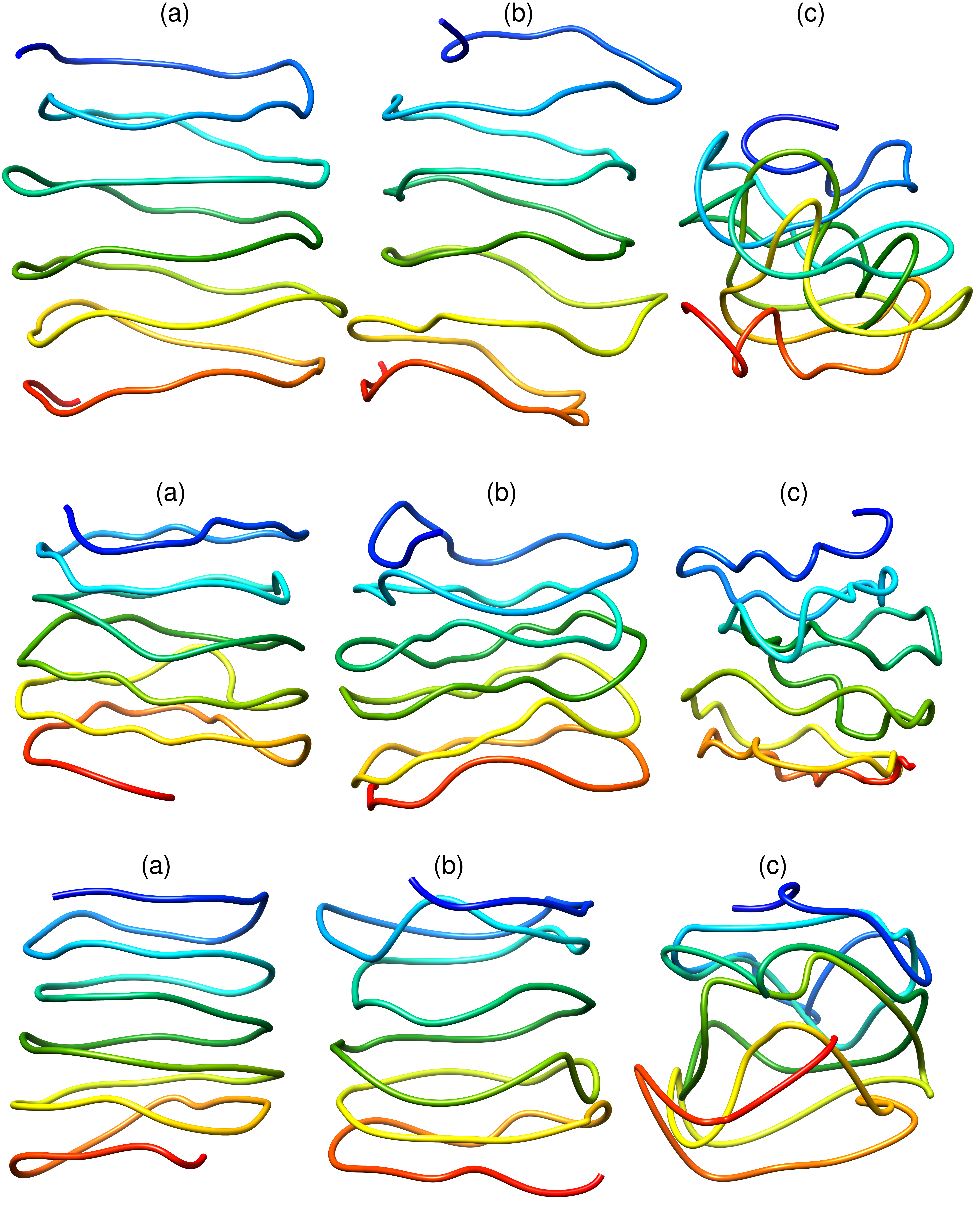


**Figure S2.** Reconstructed regular helical structure models reconstructed using Chromosome3D (top row), HSA (middle row), and Shrec3D (bottom row). The first column models (a) are the models reconstructed at 90% signal coverage, second column models (b) at 70%, and third column (c) at 25% signal coverage.
