# Supplementary material for: Chromosome3D: reconstructing three-dimensional chromosomal structures from Hi-C interaction frequency data using distance geometry simulated annealing
Source: BMC Genomics. 2016 Nov 7;17:886. doi: 10.1186/s12864-016-3210-4 (PMC5100196; doi:10.1186/s12864-016-3210-4)
Supplement: Additional file 3: Figure S3. — Top structures for all 23 pairs of chromosomes (numbered sequentially) visualized using UCSF Chimera. For each chromosome two structures are shown side by side – a structure at 1 MB resolution on the left and a structure at 500 KB resolution on the right. (DOCX 502 kb) [file 12864_2016_3210_MOESM3_ESM.docx]

# **Chromosome3D: Reconstructing Three-Dimensional Chromosomal Structures from Hi-C Interaction Frequency Data using Distance Geometry Simulated Annealing**

### Badri Adhikari^§^**,** Tuan Trieu^§^**,** Jianlin Cheng*

Computer Science Department, University of Missouri, Columbia, Missouri, 65211, USA

*Corresponding author: [chengji@missouri.edu](mailto:chengji@missouri.edu)

^§^These authors contributed equally to this work


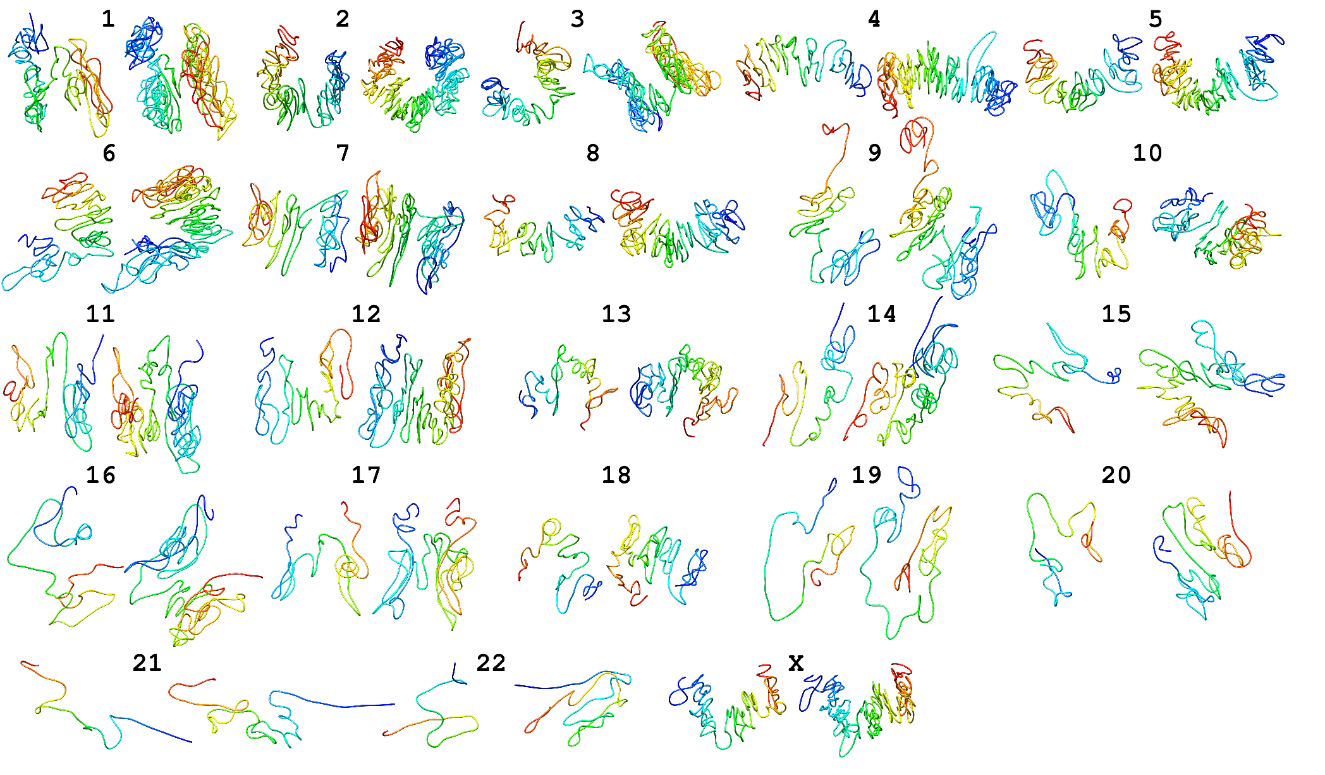


**Figure S3.** Top structures for all 23 pairs of chromosomes (numbered sequentially) visualized using USEF Chimera. For each chromosome two structures are shown side by side – a structure at 1MB resolution on the left and a structure at 500KB resolution on the right.
