# Supplementary material for: Chromosome3D: reconstructing three-dimensional chromosomal structures from Hi-C interaction frequency data using distance geometry simulated annealing
Source: BMC Genomics. 2016 Nov 7;17:886. doi: 10.1186/s12864-016-3210-4 (PMC5100196; doi:10.1186/s12864-016-3210-4)
Supplement: Additional file 5: Table S1. — Spearman’s rank correlation coefficient between distances obtained from reconstructed structures and input IFs computed after filtering 0.1 L short-range values; the numbers in parenthesis are the SRCC values for extended models used for comparison. The last two columns report the execution time in hours and minutes. (DOCX 22 kb) [file 12864_2016_3210_MOESM5_ESM.docx]

# **Chromosome3D: Reconstructing Three-Dimensional Chromosomal Structures from Hi-C Interaction Frequency Data using Distance Geometry Simulated Annealing**

### Badri Adhikari^§^**,** Tuan Trieu^§^**,** Jianlin Cheng*

Computer Science Department, University of Missouri, Columbia, Missouri, 65211, USA

*Corresponding author: [chengji@missouri.edu](mailto:chengji@missouri.edu)

^§^These authors contributed equally to this work

**Table S1** Spearman’s rank correlation coefficient between distances obtained from reconstructed structures and input IFs computed after filtering 0.1L short-range values; the numbers in parenthesis are the SRCC values for extended models used for comparison. The last two columns report the execution time in hours and minutes.

| Chromosome ID | Number of points at 500KB | SRCC | | Time in HH:MM | |
| --- | --- | --- | --- | --- | --- |
|  |  | 1MB | 500KB | 1MB | 1MB |
| 1 | 455 | -0.84 (-0.57) | -0.82 (-0.59) | 1:05 | 4:00 |
| 2 | 479 | -0.88 (-0.69) | -0.86 (-0.70) | 1:25 | 5:28 |
| 3 | 390 | -0.85 (-0.62) | -0.86 (-0.65) | 0:48 | 3:39 |
| 4 | 377 | -0.94 (-0.91) | -0.95 (-0.92) | 0:47 | 3:56 |
| 5 | 354 | -0.93 (-0.84) | -0.93 (-0.86) | 0:39 | 2:41 |
| 6 | 337 | -0.91 (-0.68) | -0.91 (-0.70) | 0:39 | 2:26 |
| 7 | 312 | -0.88 (-0.67) | -0.87 (-0.68) | 0:32 | 1:58 |
| 8 | 287 | -0.92 (-0.83) | -0.92 (-0.85) | 0:30 | 1:41 |
| 9 | 235 | -0.88 (-0.72) | -0.87 (-0.74) | 0:21 | 1:08 |
| 10 | 266 | -0.90 (-0.70) | -0.90 (-0.72) | 0:20 | 1:16 |
| 11 | 264 | -0.88 (-0.49) | -0.87 (-0.49) | 0:22 | 1:22 |
| 12 | 262 | -0.88 (-0.68) | -0.88 (-0.70) | 0:24 | 1:24 |
| 13 | 192 | -0.88 (-0.78) | -0.89 (-0.81) | 0:11 | 0:41 |
| 14 | 176 | -0.87 (-0.54) | -0.84 (-0.58) | 0:09 | 0:36 |
| 15 | 165 | -0.82 (-0.54) | -0.84 (-0.59) | 0:08 | 0:33 |
| 16 | 159 | -0.83 (-0.51) | -0.72 (-0.51) | 0:09 | 0:31 |
| 17 | 157 | -0.80 (-0.44) | -0.72 (-0.48) | 0:08 | 0:28 |
| 18 | 150 | -0.82 (-0.60) | -0.81 (-0.67) | 0:08 | 0:25 |
| 19 | 113 | -0.80 (-0.23) | -0.73 (-0.28) | 0:05 | 0:15 |
| 20 | 120 | -0.84 (-0.41) | -0.80 (-0.45) | 0:05 | 0:16 |
| 21 | 73 | -0.89 (-0.66) | -0.84 (-0.71) | 0:02 | 0:08 |
| 22 | 70 | -0.82 (-0.34) | -0.74 (-0.45) | 0:02 | 0:06 |
| X | 305 | -0.93 (-0.89) | -0.93 (-0.89) | 0:25 | 1:03 |
| Average | 248 | -0.87 (-0.62) | -0.85 (-0.65) | 0:25 | 1:34 |
