# Supplementary material for: Chromosome3D: reconstructing three-dimensional chromosomal structures from Hi-C interaction frequency data using distance geometry simulated annealing
Source: BMC Genomics. 2016 Nov 7;17:886. doi: 10.1186/s12864-016-3210-4 (PMC5100196; doi:10.1186/s12864-016-3210-4)
Supplement: Additional file 7: Figure S4. — The two compartment features highlighted in Chromosome 1 (left) and 2 (right) in the models reconstructed by Chromosome3D (top row) and PM2 (bottom row). (DOCX 761 kb) [file 12864_2016_3210_MOESM7_ESM.docx]

# **Chromosome3D: Reconstructing Three-Dimensional Chromosomal Structures from Hi-C Interaction Frequency Data using Distance Geometry Simulated Annealing**

### Badri Adhikari^§^**,** Tuan Trieu^§^**,** Jianlin Cheng*

Computer Science Department, University of Missouri, Columbia, Missouri, 65211, USA

*Corresponding author: [chengji@missouri.edu](mailto:chengji@missouri.edu)

^§^These authors contributed equally to this work


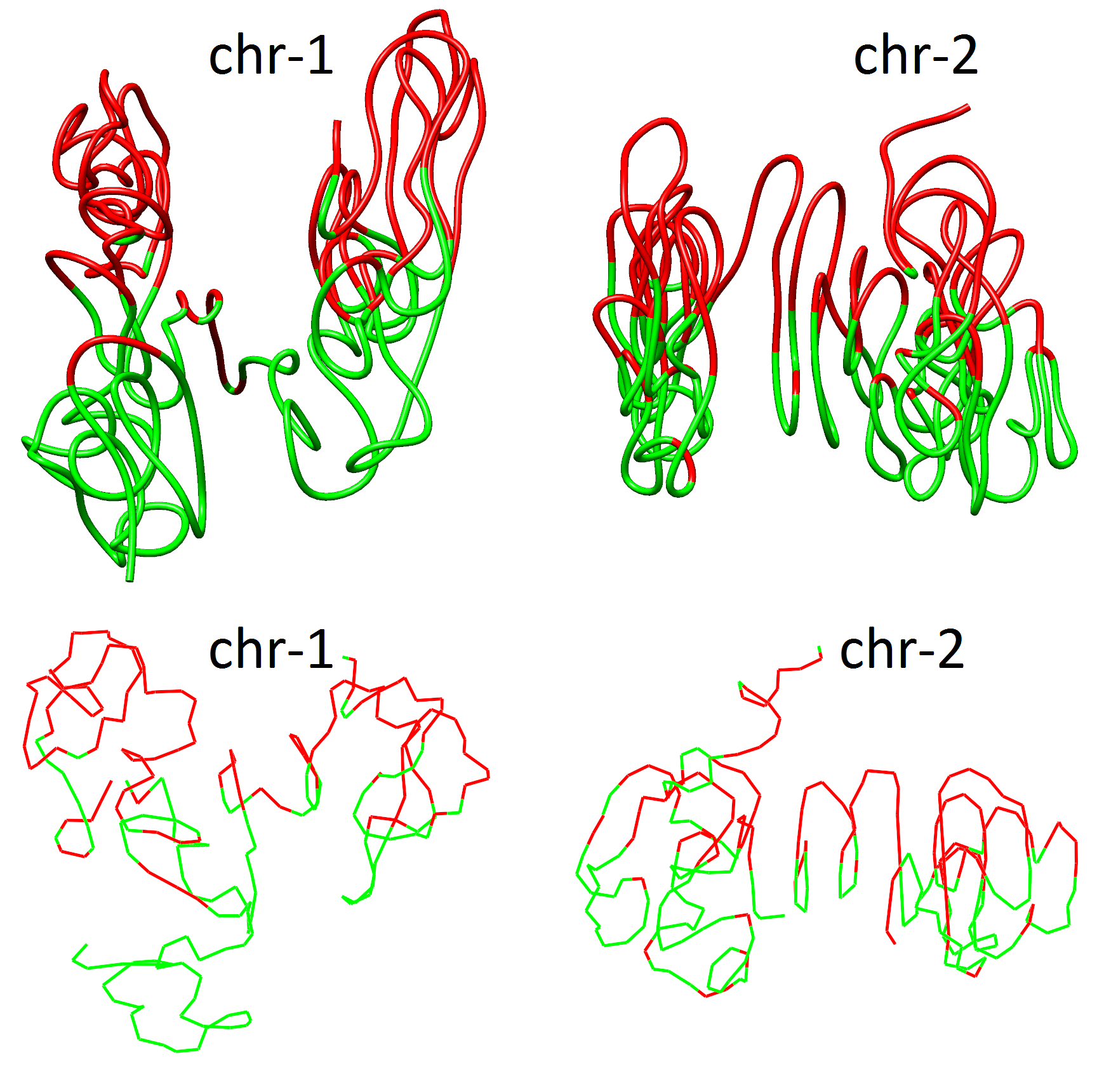


**Figure S4.** The two compartment features highlighted in Chromosome 1 (left) and 2 (right) in the models reconstructed by Chromosome3D (top row) and PM2 (bottom row).
