# Supplementary material for: Chromosome3D: reconstructing three-dimensional chromosomal structures from Hi-C interaction frequency data using distance geometry simulated annealing
Source: BMC Genomics. 2016 Nov 7;17:886. doi: 10.1186/s12864-016-3210-4 (PMC5100196; doi:10.1186/s12864-016-3210-4)
Supplement: Additional file 8: — Supplementary sections on Methods. (DOCX 1850 kb) [file 12864_2016_3210_MOESM8_ESM.docx]

# **Chromosome3D: Reconstructing Three-Dimensional Chromosomal Structures from Hi-C Interaction Frequency Data using Distance Geometry Simulated Annealing**

### Badri Adhikari^§^**,** Tuan Trieu^§^**,** Jianlin Cheng*

Computer Science Department, University of Missouri, Columbia, Missouri, 65211, USA

*Corresponding author: [chengji@missouri.edu](mailto:chengji@missouri.edu)

^§^These authors contributed equally to this work

**Supplementary Methods**

**Extended structures as reference**

We built extended or unfolded structures for each chromosome as a reference for assessing reconstructed chromosomal structures. An extended structure is a three-dimensional structure with all points placed sequentially in a line (roughly) in the Cartesian coordinate system, where adjacent points satisfy a predefined distance (or a range of predefined distances) (see **Figure 1** for an example). Such a structure can be easily built according to the number of beads without any knowledge of interaction frequency data. In eukaryotic cells with billions of base pairs packed inside a tiny nucleus, it is reasonable to assume the real chromosomal structures are quite different from extended structures. Hence, a sensible structure evaluation method that evaluates three-dimensional structures against an input IF matrix should be able to assign a lower score to such an extended structure. Using extended structures as reference allows us to check how well our evaluation method (Spearman’s rank correlation calculation) can distinguish good and bad structures.


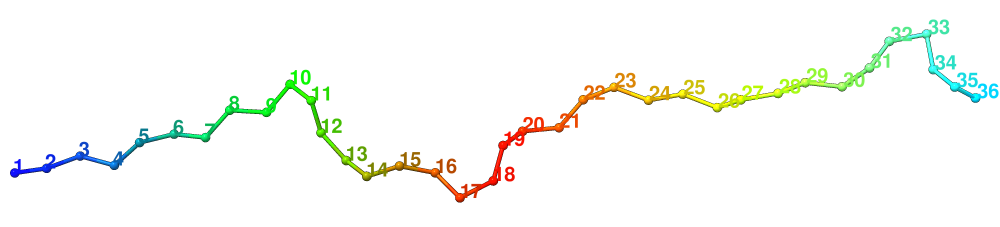


**Figure 1** An extended structure for chromosome 23 at 1MB resolution with all 36 beads/points. At 1MB resolution, each point represents 10^6^ base pairs.

**Spearman’s Rank Correlation Coefficient for structure selection**

The absence of a standard method to evaluate 3D structures against the input interaction frequency matrix imposes a major challenge to identify the chromosomal structures of good quality. Hypothesizing that the distances in better structures should have higher negative correlation with input IF values than the worse ones, we use Spearman’s rank correlation coefficient (ρ) for assessing the structures against the input IF, which was previously discussed in [1] [2]. We initially experimented computing ρ values between the triangular interaction frequency matrix flattened into a single column and the triangular distance matrix flattened into a single column. The ρ values calculated on the entire interaction frequency matrix, however, is often dominated by the majority of short-range interaction values between adjacent chromosomal regions in the IF and distance matrices (see **Figure 2**), and hence the structures realizing more short-range values will have higher correlation coefficients. For instance, although an extended structure is not folded, the pairwise distances in it still has a relatively high correlation with the input matrix because it realizes many short-range contacts. Similarly, even a random structure’s distance matrix can be highly correlated (more than 0.9) with the input IF matrix, which renders the naïve use of the correlation metric not effective for evaluating 3D chromosomal models.


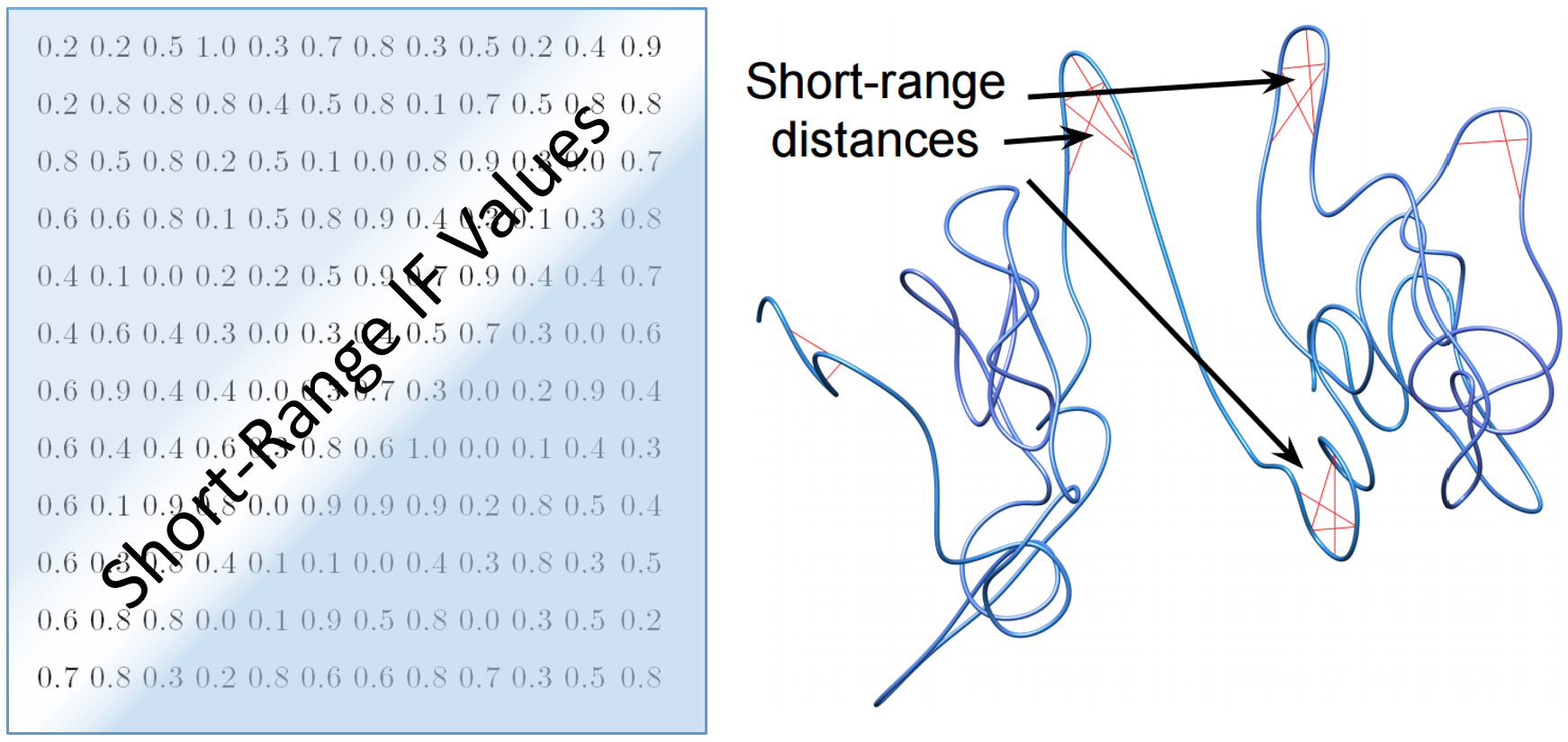


**Figure 2** Short-range contact regions along the diagonal in an interaction frequency matrix (left) and some short-range contacts in a three-dimensional structure (right). The number of values that fall in the short-range category can be defined using a sequence separation threshold. For instance, if we define $0.1L$ as the threshold for short-range data for a matrix of dimension $L$ by $L$, then all values in the cell $[i,j]$, where $\left| i-j \right|\leq0.1L$, are defined as short-range. Short-range values are large in count in a matrix because they are around the diagonal.

To verify that the high correlation between an IF matrix and a distance matrix derived from an extended structure is indeed because of the short-range values, we iteratively removed an increasing number of the short-range IF values and the corresponding distances from both matrices to compute Spearman’s rank correlation coefficient (ρ) in order to check how the correlation values changes. Specifically, in each iteration we removed all values of the matrix cells $[i,j]$ if $\left| i-j \right|\leq x * L$, where L is the size of the square matrix (row count or column count), and *x* is the fraction in the range [0, 1]. The ρ for various values of *x* for 23 pairs of human chromosome in an extended structure or a random structure are plotted in **Figure 3**. The figure shows that, without removing short-range contacts, there is a high negative correlation value, but after removal of approximately 0.3L short-range interactions, the extended structures or randomly chosen structures have relatively very low values of ρ (e.g. close to 0). Through these experiments, we hypothesize that properly folded structures should have ρ values much higher than those of extended structures.

To overcome this problem of applying Spearman’s rank correlation coefficient (ρ) to an interaction frequency matrix, we tested two approaches: **(a)** removing short-range data using a threshold to define short-range data (see **Figure 3**) and then calculating ρ, and **(b)** calculating ρ for a range of thresholds of defining short-range contacts, followed by computing mean of the coefficients. The principle idea behind the second approach is to compute correlation coefficient for many subsets with different proportions of short-range and long-range contacts, and then compute the mean of all the coefficients. Our experiment shows that the second approach is generally a stronger measure to distinguish good and bad structures than the first measure, but it takes much longer to compute. On the data sets used in this work, the first approach showed reasonable evaluations at threshold values of at least 0.1L, and hence we performed all our correlation computations by ignoring all short-range data in cell values $[i,j]$ with $\left| i-j \right|\leq0.1L$.


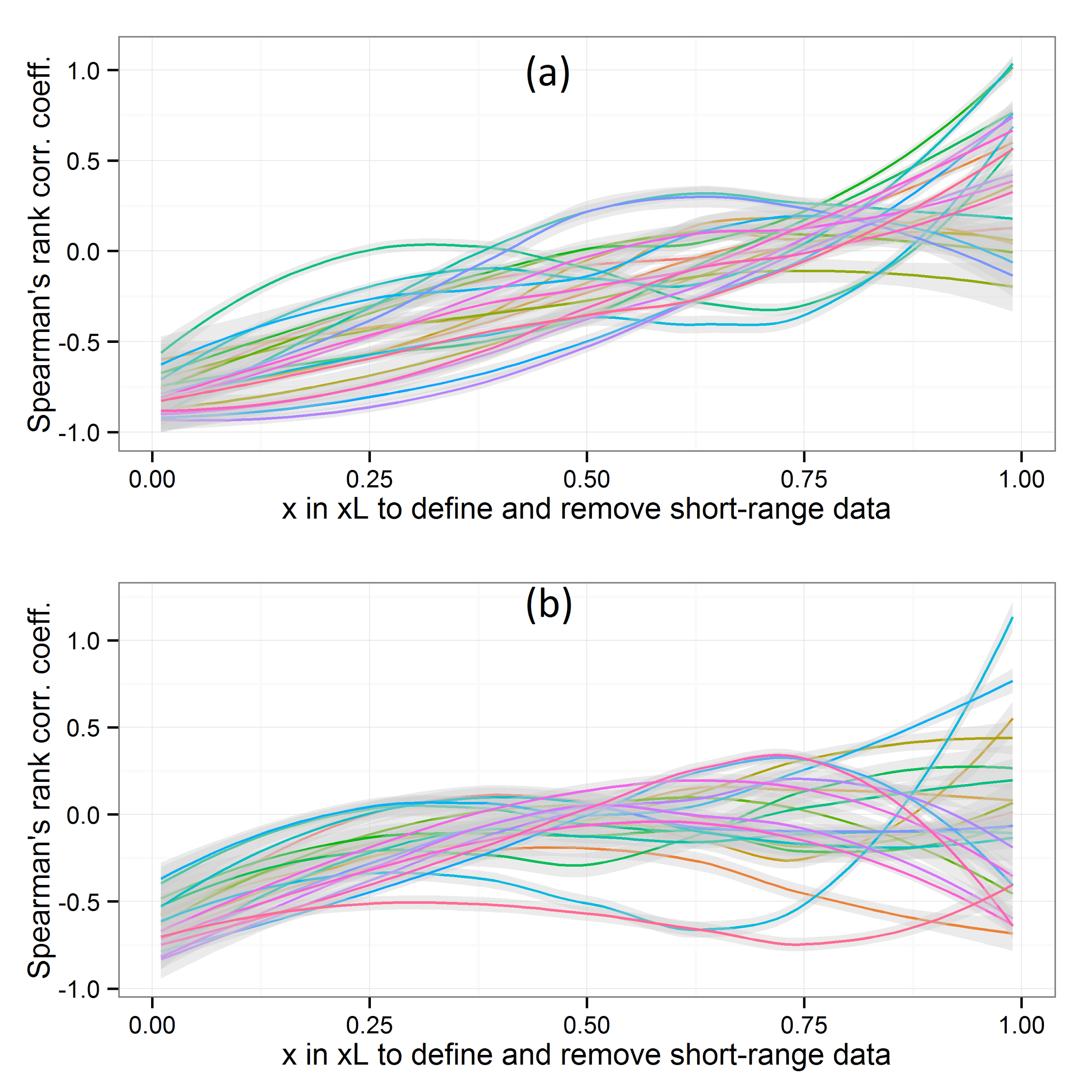


**Figure 3** Spearman’s rank correlation coefficients for all 23 chromosomes’ at 1MB resolution between IF matrices and distance matrices for extended structures **(a)** and a random structure **(b)**. The coefficients are calculated for various values of thresholds (*x*) of define short-range contacts to be ignored during correlation calculations.

**Customization of the distance geometry simulated annealing (DGSA) algorithm**

Recently many authors have broken down the problem of DNA structure reconstruction into two sub-problems – conversion of interaction frequency to distance restraints and reconstruction of 3D models from the wish distances [2] [3] [4] [5]. Our work is based on the assumption that the later sub-problem of DNA reconstruction is similar to the problem of determining protein 3D structure from a set of experimentally determined distance restraints between the atoms (i.e. a distance geometry problem) both being polymer folding problems following biophysics and biochemical principles. Hence, if we are able to translate/transform the distance restraints obtained from interaction frequency into distances that are in similar range and units as protein distance restraints, the algorithms that are established for protein structure determination can be applied and tested on DNA restraints.

We customized the default distance geometry simulated annealing (DGSA) protocol implemented in the script ‘dg_sa.inp’ in CNS suite by adjusting some parameters to fit chromosomal structure modeling as follows. To speed up the reconstruction, we updated the atom selection parameter by setting ‘par.1’ to ‘protein.param’ instead of ‘protein-allhdg.param’ to ignore all hydrogen atoms. Because noisy and conflicting restraints often result in too compact models, we increased the initial and final radius parameters ‘md.cool.init.rad’ and ‘md.cool.fina.rad’ to 1.0 and 0.85 respectively in order to increase repulsion between units, and reduced the weight of the distance restraints by decreasing both parameters ‘md.cool.noe’ and ‘md.pow.noe by five times and setting their values to 10. Considering the large number of restraints and chromosomal units, the number of minimization steps is increased 75 times by setting the parameter ‘md.pow.step’ to 15000. We also doubled the model count to 20 by updating the parameters ‘pdb.dg.count’ and ‘pdb.end.count’. Finally, we increased the number of restraints that the DGSA protocol can accept by 10 times through setting the ‘nrestraints’ parameter in the ‘readdata’ module to 20000.

**Preparation of Noisy simulated dataset**

The noise-free IF matrix was used as the first simulated dataset. We then introduced noise into the IF matrix to make 12 additional datasets with different levels of noise by the following procedure: $\beta\%$ of pairs of contacts were randomly selected without replacement, and for each pair of contacts, their $IF$ values ($IF_{ij}$ and $IF_{kl}$) were adjusted as follows, $IF_{kl}=IF_{kl}+\alpha*IF_{ij}$ and $IF_{ij}=IF_{ij}-\alpha*IF_{ij}$ , where $\alpha$ is a random number in the range [0.0, 0.5] and $\beta\%$ varies between 3% and 40% (i.e. 3%, 5%, 7%, 10%, 13%, 15%, 17%, 20%, 25%, 30%, 35%, and 40%). Converting these noisy IFs into wish distances will produce distorted distances from the true distances and result in inconsistent constraints that can simulate inconsistent constraints in Hi-C data.

**References**

[1] N. Varoquaux, F. Ay, W. S. Noble, and J.-P. Vert, “A statistical approach for inferring the 3D structure of the genome,” *Bioinformatics*, vol. 30, no. 12, pp. i26–i33, Jun. 2014.

[2] Z. Zhang, G. Li, K.-C. Toh, and W.-K. Sung, “3D chromosome modeling with semi-definite programming and Hi-C data,” *J. Comput. Biol. J. Comput. Mol. Cell Biol.*, vol. 20, no. 11, pp. 831–846, Nov. 2013.

[3] M. Rousseau, J. Fraser, M. A. Ferraiuolo, J. Dostie, and M. Blanchette, “Three-dimensional modeling of chromatin structure from interaction frequency data using Markov chain Monte Carlo sampling,” *BMC Bioinformatics*, vol. 12, p. 414, 2011.

[4] Z. Duan, M. Andronescu, K. Schutz, S. McIlwain, Y. J. Kim, C. Lee, J. Shendure, S. Fields, C. A. Blau, and W. S. Noble, “A three-dimensional model of the yeast genome,” *Nature*, vol. 465, no. 7296, pp. 363–367, May 2010.

[5] A. Lesne, J. Riposo, P. Roger, A. Cournac, and J. Mozziconacci, “3D genome reconstruction from chromosomal contacts,” *Nat. Methods*, vol. 11, no. 11, pp. 1141–1143, Nov. 2014.
